# Effects of two inhibitors of metabolic glutamate receptor 5 on expression of endogenous homer scaffold protein 1 in the auditory cortex of mice with tinnitus

**DOI:** 10.1080/21655979.2021.1979354

**Published:** 2021-09-21

**Authors:** Weiwei Yan, Hongfei Zhu, Bianbian Yu, Xin Ma, Hang Liang, Shuyan Zhao, Kebin Deng

**Affiliations:** a The First Clinical College, Hubei University of Chinese Medicine, Wuhan, Hubei, China; b Department of Anesthesiology, Hubei Provincial Hospital of Traditional Chinese Medicine, Wuhan, Hubei, China; c Department of Otorhinolaryngology, Hubei Provincial Hospital of Traditional Chinese Medicine, Wuhan, Hubei, China

**Keywords:** Auditory cortex, homer1, metabolic glutamate receptor 5 antagonists, tinnitus

## Abstract

Tinnitus is deemed as the result of abnormal neural activities in the brain, and Homer proteins are expressed in the brain that convey nociception. The expression of Homer in tinnitus has not been studied. We hypothesized that expression of Homer in the auditory cortex was altered after tinnitus treatment. Mice were injected with sodium salicylate to induce tinnitus. Expression of Homer was detected by quantitative real-time polymerase chain reaction, western blotting, and immunohistochemistry assays. We found that Homer1 expression was upregulated in the auditory cortex of mice with tinnitus, while expression of Homer2 or Homer3 exhibited no significant alteration. Effects of two inhibitors of metabolic glutamate receptor 5 (mGluR5), noncompetitive 2-Methyl-6-(phenylethynyl)-pyridine (MPEP) and competitive α-methyl-4-carboxyphenylglycine (MCPG), on the tinnitus scores of the mice and on Homer1 expression were detected. MPEP significantly reduced tinnitus scores and suppressed Homer1 expression in a concentration dependent manner. MCPG had no significant effects on tinnitus scores or Homer1 expression. In conclusion, Homer1 expression was upregulated in the auditory cortex of mice after tinnitus, and was suppressed by noncompetitive mGluR5 inhibitor MPEP, but not competitive mGluR5 inhibitor MCPG.

## Introduction

Tinnitus, with the prevalence of 10%–15% [1], refers to the perception of sound without external sound stimulus and is usually accompanied by hearing loss. Tinnitus is distinguished from auditory hallucinations, the latter one involving the symptoms of hearing one or more talking voices. Around 1–2% of global population was interfered by severe tinnitus [1]. In some cases, social isolation, depression, and suicide may be caused by tinnitus [2]. The prevalence of sound-induced tinnitus is on the increase, especially in young people with the increased intake of white bread, carbonated beverages, and fast food [3].

Tinnitus is perceived as originating from cochlea [4], and the central nervous system [5]. Some studies revealed that tinnitus is a conscious percept and is influenced by emotion, attention, memory, and learning [1,6,7]. Functional neural imaging studies in patients with tinnitus showed alterations in the classical auditory pathway and the cortical regions associated with attention, emotion, memory, and perception functions [8–11]. Therefore, tinnitus is deemed as the result of abnormal neural activities in some trigger zones of the brain, causing a series of changes in brain networks [6].

The Homer proteins are products of three mammalian genes (Homer1-3), one Xenopus gene and one Drosophila gene [12]. Homer proteins are similar in structure including a conserved amino-terminal EVH1 domain that binds to group1 metabotropic glutamate receptors (mGluRs) [13], ryanodine receptors [14], inositol-1,4,5-triphosphate receptors [13], and transient receptor potential canonical-1 ion channels [15]. Homer proteins are expressed in brain regions that convey nociception [16,17]. Homer1 is localized to chromosome 5 (5q14.2) and encodes many transcriptional variants in humans. Homer1 (but not Homer2) knockout leads to glutamate abnormalities in prefrontal cortex, providing evidence for the selective disadvantageous effects of Homer1 silencing on emotional, sensorimotor, cognitive, and motivational processing [18]. Considering the close relationship between tinnitus and attention, emotion, memory, perception functions, we hypothesized that Homer1 is associated with tinnitus. Homer1 proteins bind to mGluR5 [19]. 2-Methyl-6-(phenylethynyl)-pyridine (MPEP; formula: C₁₄H₁₁N) is a potent, noncompetitive, orally active, and systemically active mGluR5 antagonist, while α-methyl-4-carboxyphenylglycine (MCPG; formula: C₁₀H₁₁NO₄) is a phenylglycine derivative and a competitive mGluR5 antagonist. Both MPEP and MCPG have been suggested to have antidepressant-like effects in rodents [20,21].

Salicylate can induce aberrant excitability of central and peripheral auditory neurons [22]. Rodents injected with salicylate were commonly used for tinnitus research [23–25]. We made a hypothesis that tinnitus is related to the abnormal expression of Homer genes in the auditory cortex. After establishing a salicylate-stimulated tinnitus mouse model, expression of Homer genes in the auditory cortex was detected and we identified upregulated expression of Homer1, but not of Homer2 or Homer3 in auditory cortex of mice after salicylate stimulation. The effects of two mGluR5 inhibitors, MPEP and MCPG, on tinnitus scores and on expression of Homer1 were subsequently investigated.

## Materials and methods

### Animals and grouping

Because tinnitus is most prevalent in older humans, we used the senescence-accelerated mouse-prone 8 (SAMP8) mice in the present study, which is consistent with the previous studies [26,27]. Male SAMP8 mice (three-month-old; 22–33 g) were randomly divided into 8 groups: saline (n = 10), 300 mg/kg salicylate (n = 20) [26], salicylate + 100 nmol/L of MPEP (n = 20), salicylate + 250 nmol/L of MPEP (n = 20), salicylate + 300 nmol/L of MPEP (n = 20), salicylate + 100 nmol/L of MCPG (n = 20), salicylate + 250 nmol/L of MCPG (n = 20), salicylate + 300 nmol/L of MCPG (n = 20). Concentrations of MPEP and MCPG were determined according to our preliminary assays. Animal experiments were approved by the Institutional Animal Care and Use Committee of Hubei Provincial Hospital of Traditional Chinese Medicine (Hubei, China).

### Conditioning paradigm

The conditioning to the task is composed of 6 sessions that were performed daily for 5 days (days 1–5) [26]. There were 10 trials for each session that lasts 15–20 min with the intervals of at least 1 min. The conditioned stimulus was a 50 dB pure tone (frequency: 10 kHz; duration: 3 seconds), while the unconditioned stimulus was an electric foot-shock (3.7 mA; duration: no more than 30 seconds) according to Guitton’s protocols [28]. The interval between the conditioned and the unconditioned stimuli was 1 second. After the coupled conditioned and unconditioned stimulus, mice climbed the pole to a safe area. When the animal climbed correctly, the electrical shocks would be stopped. The ‘truepositive’ score was evaluated according to times the mice climbed correctly in response to sound. When the score reached at least 80% in 3 continuous sessions, mice were considered as conditioned, and were used for the tinnitus experiments.

### Induction and testing of tinnitus

Upon conditioned, the mice had a rest for 1 day (day 6). On the next 4 consecutive days (days 7–10), an active avoidance task was conducted 2 h after intraperitoneal injections of saline alone or that containing sodium salicylate (300 mg/kg; Sigma, St. Louis, MO, USA) [26]. The active avoidance task included 10 trials. To compensate for hearing loss induced by salicylate, the intensity of sound that produced the behavioral responses was increased to 70 dB for mice in the salicylate-treated group to make sure that mice in both groups had similar sound sensation levels. During testing, 3-second sound was given to the mice. After that, mice were observed to see whether they would climb to the safe area as conditioned in 5 seconds (true-positive). If the mice stayed in the safe area for more than 10 seconds, they would be returned to the floor for following observation, otherwise, mice will be given with electrical shocks until they climbed to the safe area. Finally, the total number of times that the mice climbed during the inter-trial silent period of 1 minute for 10 trials were calculated as the tinnitus scores.

### Tissue preparation and combined in situ hybridization and immunohistochemistry

Auditory cortices were identified according to a previous study [29]. Tissues within the anterior and posterior ectosylvian sulci (roughly delineating A1) were dissected. RNA was isolated from tissues as a previous study described [30]. Combined in situ hybridization and immunohistochemistry method was used for detection of existence of Homer1 mRNA and protein in auditory cortex according to a previous study [31]. The primary antibody against Homer1 protein (1:500, ab184955) and the secondary antibody (1:100, ab7090) used in the immunohistochemistry was purchased from Abcam.

### Quantitative real-time polymerase chain reaction (qRT-PCR)

Total RNA was extracted from auditory cortex tissue using a TRIzol™ Plus RNA Purification Kit (Invitrogen, CA, USA). Concentration and purity of RNA were evaluated using a photometer (NanoDrop 8000, Thermo, USA). Extracted RNAs were reverse transcribed to cDNA using a PrimeScript RT reagent Kit (Takara, Dalian, China) at 25°C for 5 min, 50°C for 45 min. Inactivation of the reverse transcriptase was performed by heating to 85°C for 5 min. The mRNA expression levels were calculated by the 2^−ΔΔCt^ method [32] and were normalized to Gapdh gene expression. Relative primer sequences used for PCR were listed as follows: Homer1, forward (F), 5ʹ-CAGGAATCAGCAGGAGGAG-3ʹ, reverse (R), 5ʹ-GATTGCTGAACTAGCATGAGAG-3ʹ; Homer2, F, 5ʹ-AGGAGATGGAGTTGAAAGATCTC-3ʹ, R, 5ʹ-AGGAGATGGAGTTGAAAGATCTC-3ʹ; Homer3, F, 5ʹ-TCAGGAGGTGAAAGAAGCTG-3ʹ, R, 5ʹ-CTTGGAGGAACCTGATGGG-3ʹ; Gapdh, F, 5ʹ-CATCTTCTTGTGCAGTGCC-3ʹ, R, 5ʹ-CAAATCCGTTCACACCGAC-3ʹ.

### Western blotting analysis

Tissue samples were lysed using RIPA buffer (Beyotime, Shanghai, China) with protease inhibitor cocktail (Sigma). A Bicinchoninic Acid Protein Assay Kit (Thermo Fisher) was used for protein quantification. The same amount (50 μg/lane) of protein was separated from SDS-PAGE and blotted onto PVDF membranes followed by incubation with the primary antibodies against Homer1 (1/1000; ab184955; Abcam), Homer2 (1/1000; PA5-70,541; Invitrogen), Homer3 (1/2000; PA5-79,384; Invitrogen), GAPDH (1/2500; ab9485; Abcam) overnight at 4°C. Next, samples were incubated with the secondary antibodies at room temperature for 2 h. GAPDH served as an internal control. Protein visualization was performed with an ECL chemiluminescence kit (Thermo Fisher). The ImageJ version 1.8.0 software (National Institutes of Health) was used to quantify each protein band.

## Statistical analysis

Statistical analysis was conducted using SPSS 20 software. GraphPad Prism 7 was used for graph drawing. Student’s *t* test was performed to analyze the difference between 2 groups, while one-way or two-way analysis of variance was used for difference analysis among 3 or more groups. Correlation between tinnitus scores and expression level of Homer1 was assessed by linear regression analysis. *P* values less than 0.05 were defined significant.

## Results

### Effects of salicylate on tinnitus scores, body weight, and midbrain weight in mice

**Figure 1.**
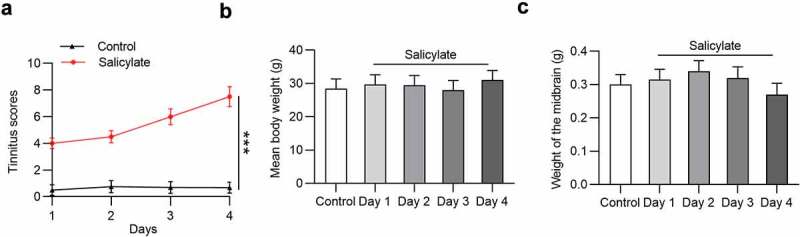
**The daily tinnitus scores, weight of body and midbrain of mice in the control group and the salicylate group**. (a) Tinnitus scores of mice after saline or salicylate injection for 4 days. (b) Body weight of mice in the control group and the salicylate group after injections of saline or salicylate for 4 days. (c) Weight of midbrain of mice after injections of saline or salicylate for 4 days. ***p < 0.001

Effects of salicylate on tinnitus scores, body weight, and midbrain weight in mice were detected. The tinnitus scores were not elevated after injection of normal saline into mice in the following 4 days, but were significantly increased in salicylate-treated mice in a day-dependent manner (Figure 1a). The mean tinnitus score of mice after salicylate stimulation for 4 days was increased by 10.87 folds compared to that for 1 day. No significant difference was found in the mean body weight (Figure 1b) and midbrain weight (Figure 1c) of mice between the control group and the salicylate group.

### Changes of homer expression in the auditory cortex of mice after salicylate stimulation

**Figure 2.**
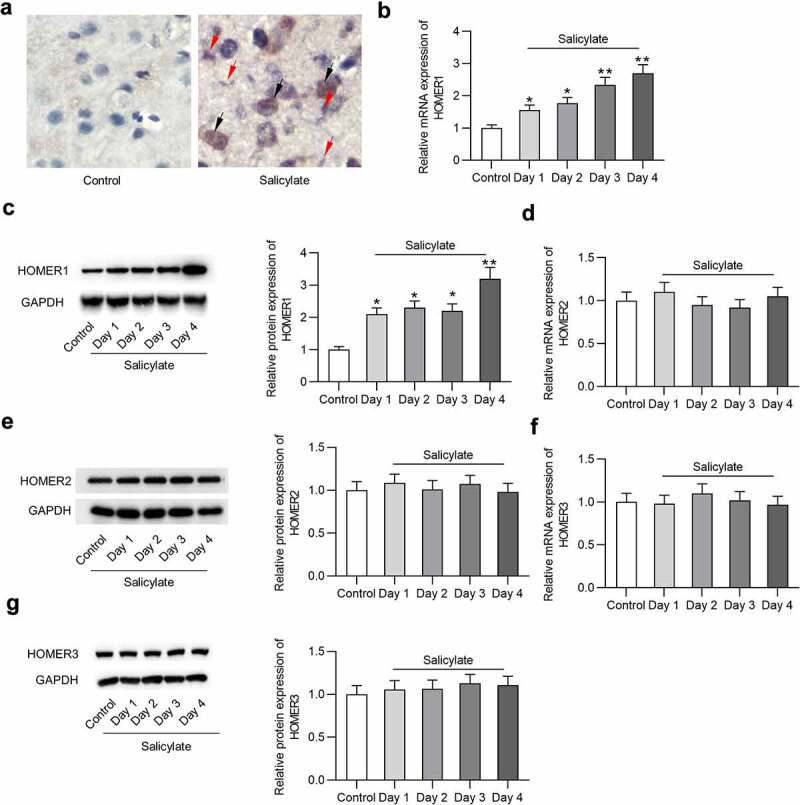
**Expression of Homer1/2/3 in the auditory cortex of mice**. (a) Homer1 mRNA and protein is expressed in the auditory cortex (temporal cortex area 3) of mice in the salicylate group. Scale bar: 500 μm. Red arrow points to Homer1 protein (bluish violet) and black arrow points to Homer1 mRNA (claybank). (b) Relative Homer1 mRNA expression in the auditory cortex of mice after injections of saline or salicylate on Day 1, Day 2, Day 3, Day 4 was detected by PCR analysis. (c) Relative Homer1 protein expression in the auditory cortex of mice after injections of saline or salicylate on Day 1, Day 2, Day 3, Day 4 was detected by western blotting analysis. (d-e) Relative Homer2 expression at the mRNA and protein levels in the auditory cortex of mice in the control group and the salicylate group on Day 1, Day 2, Day 3, Day 4 was detected by PCR and western blotting analysis. (f-g) Relative Homer3 expression at the mRNA and protein levels in the auditory cortex of mice in the control group and the salicylate group on Day 1, Day 2, Day 3, Day 4 was detected by PCR and western blotting analysis. *p < 0.05, **p < 0.01

Effects of salicylate on Homer expression in the auditory cortex of mice after salicylate stimulation were detected. Figure 2a reveals the existence of Homer1 mRNA and protein in the auditory cortex of mice *ex vivo*. Figure 2b-c reveals that salicylate induced the upregulation of Homer1 mRNA (by 2.7 folds) and protein (by 3.2 folds) expression in the auditory cortex of mice in a day-dependent manner. We also found a positive correlation between tinnitus scores and expression level of Homer1 (coefficient: 5.22; standard error: 0.3; 95% confident interval: 4.75–5.15; *P* < 0.001). Interestingly, expression of Homer2 (Figure 2d-e) or Homer3 (figure 2f-g) in the auditory cortex was not significantly affected by salicylate.

### Effects of MPEP and MCPG on tinnitus scores, body weight, and midbrain weight of salicylate-treated mice

**Figure 3.**
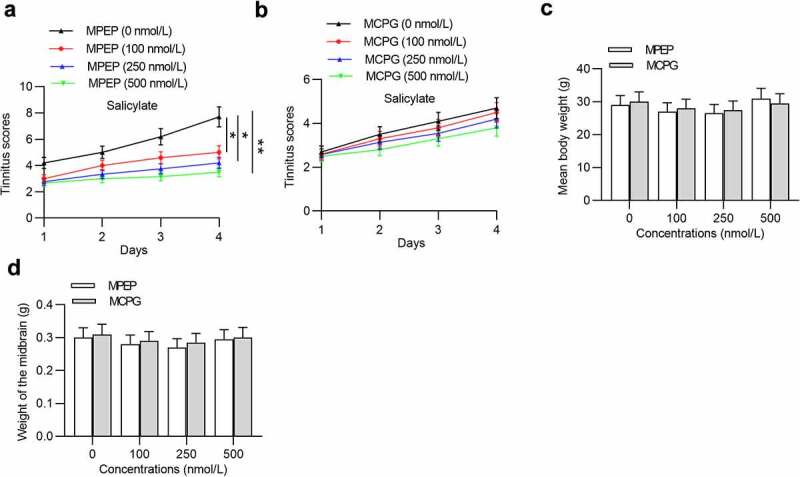
**Effects of MPEP and MCPG on the tinnitus scores, weight of body and midbrain of mice in the salicylate group**. (a) Daily tinnitus scores of mice injected with salicylate combined with different concentrations of MPEP (0, 100, 250, 500 nmol/L). (b) Daily tinnitus scores of mice injected with salicylate combined with different concentrations of MCPG (0, 100, 250, 500 nmol/L). (c) Body weight of mice injected with salicylate combined with different concentrations of MPEP (0, 100, 250, 500 nmol/L) was evaluated on the 4^th^ day after injections. (d) Body weight of mice injected with salicylate combined with different concentrations of MCPG (0, 100, 250, 500 nmol/L) was evaluated on the 4^th^ day after injections. *p < 0.05, **p < 0.01

Next, we investigated the influence of MPEP and MCPG on tinnitus scores, body weight, and midbrain weight of mice after injection with salicylate. Injection of MPEP significantly reduced tinnitus scores in a concentration-dependent manner (Figure 3a), while injection of MCPG had no significant effects on tinnitus scores (Figure 3b). Moreover, neither MPEP nor MCPG can induce significant changes in the mean body weight (Figure 3c) and midbrain weight (Figure 3d).

### Effects of MPEP and MCPG on Homer1 expression in the auditory cortex of mice

**Figure 4.**
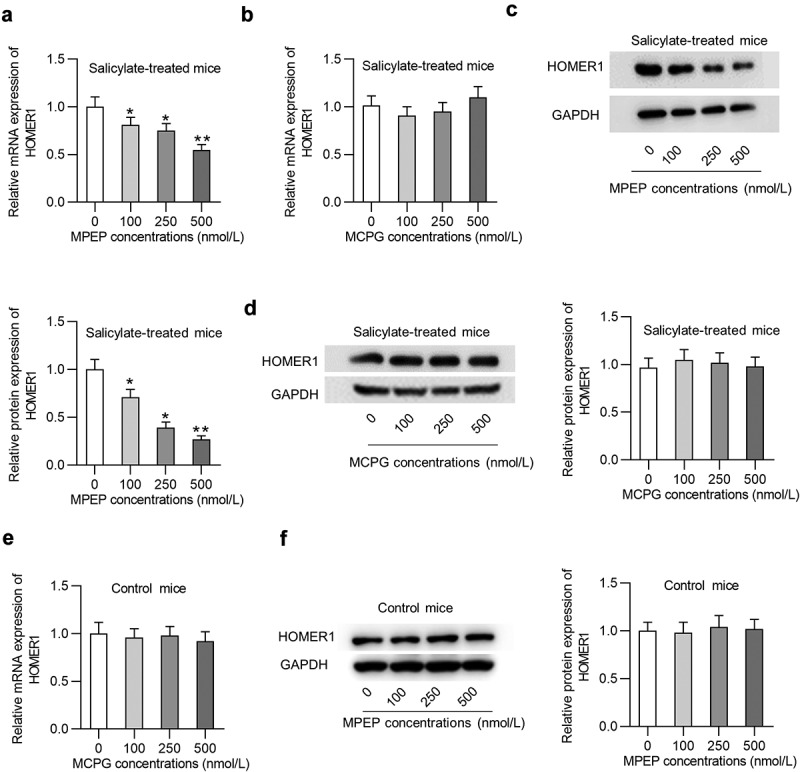
**Homer1 expression was inhibited by MPEP but not MCPG in the auditory cortex of mice in the salicylate group**. (a) Relative mRNA expression of HOMER1 in the auditory cortex of mice injected with salicylate combined with different concentrations of MPEP (0, 100, 250, 500 nmol/L). (b) Relative mRNA expression of HOMER1 in the auditory cortex of mice injected with salicylate combined with different concentrations of MCPG (0, 100, 250, 500 nmol/L). (c) Relative protein expression of HOMER1 in the auditory cortex of mice injected with salicylate combined with different concentrations of MPEP (0, 100, 250, 500 nmol/L). (d) Relative protein expression of HOMER1 in the auditory cortex of mice injected with salicylate combined with different concentrations of MCPG (0, 100, 250, 500 nmol/L). (e-f) Relative mRNA and protein expression of HOMER1 in the auditory cortex of mice injected with saline combined with different concentrations of MPEP (0, 100, 250, 500 nmol/L) were detected by PCR and western blotting analysis. *p < 0.05, **p < 0.01

Whether MPEP and MCPG regulate Homer1 expression in the auditory cortex of mice was explored. Injection of MPEP significantly reduced the mRNA expression of Homer1 (Figure 4a) in the auditory cortex of mice in the salicylate group in a concentration-dependent manner (decreased by 45% by 500 nmol/L of MPEP), while injection of MCPG had no significant effects on the mRNA expression of Homer1 (Figure 4b). Similarly, Homer1 protein levels were concentration-dependently decreased by MPEP in the auditory cortex of mice in the salicylate group (Figure 4c, decreased by 73% by 500 nmol/L of MPEP) and were not impacted by MCPG (Figure 4d). MPEP suppresses Homer1 expression at the mRNA and protein levels in the auditory cortex of control mice, indicating that the MPEP-medaited degradation of Homer1 is independent of salicylate (Figure 4e-f).

## Discussion

We confirmed tinnitus induction in mice after injection of salicylate. The present study revealed that Homer1 expression at the mRNA and protein levels was day-dependently upregulated in the auditory cortex of salicylate-treated mice, while Homer2 or Homer3 expression showed no significant alteration in response to salicylate, indicating that salicylate-induced tinnitus may result from downregulation of the Homer1 gene in the autitory cortex. Tinnitus can be caused by noise, and Homer1 was reported to be significantly downregulated in the ventral cochlear nucleus of mice 2 days after exposure to noise [33]. According to previous studies, abnormal expression of Homer1 can be induced by many external stimuluses, for example, exposure to novel environments induces the upregulation of Homer1 in cortical structures including the frontal cortex [34]. Psychotomimetic drugs induce Homer1 upregulation in several limbo-corticostriatal structures [35–37] and antipsychotic medications [38,39]. Moreover, a previous study revealed that Homer1 has the same regulatory elements with miR-96 and miR-1271, and the mutation of miR-96 and miR-1271 leads to deafness in humans and mice [40], which indicates the close association between Homer1 and hearing-related disease.

mGluRs can mediate ionotropic glutamate receptors, neurotransmitter release and various calcium and potassium channels [41] that are involved in the pathophysiology of central nervous system injury. mGluRs are categorized into group I, group II, and group III. A previous study revealed that mice mimicking human syndromic deafness exhibited impaired glutamate uptake in the cochlear nuclei [42]. mGluR5 belongs to group I and consists of heptahelical domain, cysteine-rich region, and binding domain [43]. The role of mGluR5 is controversial due to lacking authentic selective antagonists. Selective mGluR5 inhibitors including noncompetitive MPEP [44] and competitive MCPG [45] have been identified. We identified that injection of MPEP significantly reduced tinnitus scores in mice in a concentration-dependent manner, while injection of MCPG had no significant effects on tinnitus scores. Importantly, MPEP reduced Homer1 mRNA expression and protein levels in the auditory cortex of tinnitus mice, while MCPG had no significant effects on Homer1 expression. These findings indicated that MPEP, as a mGluR5 inhibitor, has an inhibitory effect on tinnitus by suppressing Homer1. Tinnitus has been reported to be highly associated with gene expression of NMDA receptor [27], and mGluR5 positive modulators enhance NMDA receptor activation [46]. MPEP can function as a weak NMDA antagonist [47]. There is also evidence for a functional interaction between mGluR5 and NMDA receptors [48]. We hypothesized that MPEP suppresses tinnitus also via inhibiting NMDA receptor expression and/or its functions.

However, some limitations of our study must be addressed. Despite that sodium salicylate at the dose of 350 mg/kg used in the present study is widely applied in rodent experiments on tinnitus, there are some side reactions such as the systemic toxicity and peripheral deafness [49]. Our findings only provided the primary evidence for the expression of Homer1 in sodium salicylate-induced tinnitus in mice, while experiments using neurons are needed to confirm the results.

## Conclusion

Our findings innovatively demonstrated that Homer1 is upregulated in the auditory cortex of mice with salicylate-induced tinnitus. A noncompetitive mGluR5 antagonist, MPEP, can reduce tinnitus scores of mice and suppress the expression of Homer1 in the auditory cortex in a concentration-dependent manner, while a competitive mGluR5 antagonist, MCPG, exerted no significant effects. Our findings could contribute to further understanding of pathophysiology and therapy of tinnitus, while more studies are needed to reveal the detailed mechanisms linking Homer1 and tinnitus in the future.
